# Analyte Quantity Detection from Lateral Flow Assay Using a Smartphone

**DOI:** 10.3390/s19214812

**Published:** 2019-11-05

**Authors:** Kamrul H. Foysal, Sung Eun Seo, Min Ju Kim, Oh Seok Kwon, Jo Woon Chong

**Affiliations:** 1 Department of Electrical & Computer Engineering, Texas Tech University, Lubbock, TX 79409, USA; kamrul.foysal@ttu.edu; 2 Infectious Disease Research Center, Korea Research Institute of Bioscience and Biotechnology (KRIBB), 125 Gwahak-ro, Yuseong-gu, Daejeon 34141, Korea; eun93618@gmail.com (S.E.S.); mjkim4427@gmail.com (M.J.K.); 3 Nanobiotechnology and Bioinformatics (Major), University of Science & Technology (UST), 125 Gwahak-ro, Yuseong-gu, Daejeon 34141, Korea

**Keywords:** LFA pad, analyte detection, smartphone, LFA reader

## Abstract

Lateral flow assay (LFA) technology has recently received interest in the biochemical field since it is simple, low-cost, and rapid, while conventional laboratory test procedures are complicated, expensive, and time-consuming. In this paper, we propose a robust smartphone-based analyte detection method that estimates the amount of analyte on an LFA strip using a smartphone camera. The proposed method can maintain high estimation accuracy under various illumination conditions without additional devices, unlike conventional methods. The robustness and simplicity of the proposed method are enabled by novel image processing and machine learning techniques. For the performance analysis, we applied the proposed method to LFA strips where the target analyte is albumin protein of human serum. We use two sets of training LFA strips and one set of testing LFA strips. Here, each set consists of five strips having different quantities of albumin—10 femtograms, 100 femtograms, 1 picogram, 10 picograms, and 100 picograms. A linear regression analysis approximates the analyte quantity, and then machine learning classifier, support vector machine (SVM), which is trained by the regression results, classifies the analyte quantity on the LFA strip in an optimal way. Experimental results show that the proposed smartphone application can detect the quantity of albumin protein on a test LFA set with 98% accuracy, on average, in real time.

## 1. Introduction

Lateral flow assay (LFA) [1] strips are widely used for analyte detection in a given liquid sample. This technology has rapidly gained interest nowadays due to its low-cost, simple, and swift detection of biochemical compounds in samples [2,3,4]. Specifically, LFA pads are cellulose-based strips containing specific reagents which are activated when the target analyte is present in the sample. LFA has been adopted in various fields of study, e.g., point of care [5,6], agriculture [7], food [8], medicine, and environmental science [9]. Moreover, LFA is important for diagnosis, such as pregnancy, heart failure [10], contamination [11], and detection of drugs of abuse [12]. Some of these LFAs are particularly designed for point of care testing [13,14].

Existing analyte detection methods using LFA strips can be categorized into qualitative and quantitative approaches [15]. Qualitative analysis is performed by assessing colors at the region of interest (ROI) visually [13]. This rapid result of qualitative tests helps make immediate decisions. However, qualitative analysis provides subjective judgement and may give a wrong interpretation [16]. Hence, quantitative methods are needed where the results are not clear with qualitative analysis. Quantitative analysis of LFA is shown to facilitate point-of-care testing compared to qualitative analysis [16,17,18,19]. Quantitative approaches require a method that can readily deliver quantitative measurements in a fast and accurate way. Due to their superior processing ability and portability, smartphones are being used for acquiring and processing biomedical signals and images [20,21,22]. Existing quantitative analysis requires external devices [23,24] in addition to a smartphone, such as an external lens, LFA housing device, or LED strip/optical fiber. Standalone devices [25] adopt specific processors, light sources, and housing for the LFA strip and are configured to exclusively perform analyte quantity detection [26,27]. For automated quantification and rapid decision, a mobile camera–based LFA detection method has been highlighted, which adopts specific lighting conditions [28] or external housing attached to a smartphone [23] for LFA detection.

For quantification of the result, optical strip readers are commonly attached to a smartphone. Images of the strips are captured with the smartphone camera, and then the optical strip reader processes the image using image processing software that does, for example, threshold-based color segmentation [29] to quantitatively measure the amount of analyte present in the sample. This procedure requires placing the LFA and light source precisely when the smartphone captures the image and also needs proper illumination for high contrast between ROI and background, which is not always easy to achieve [30]. In order to provide more accurate quantitative analysis, an LFA analyte detection system that is resilient at diverse illumination conditions needs to be developed [4]. A smartphone-based automated method quantifying the quantity of the analyte present in a sample can be promising due to its portability and ease-of-use. 

Several techniques have been adopted for analyte detection in LFA, and recent smartphone-based detection techniques are highlighted. Carrio et al. [12] and Cooper et al. [28] demonstrated that smartphone-based LFA detection can be applied to the quantification of drugs of abuse. However, these methods require devices other than a smartphone. In this paper, we propose a novel smartphone-based analyte quantity detection method for an LFA strip, which detects the amount of analyte in an LFA strip using a smartphone without an external device. We also design the proposed method to be resilient at diverse lighting conditions. Using optical methods, the application can easily detect test line and control line pixels’ intensity values without using an external strip reader device, which increases portability and convenience. Unlike a standalone LFA strip reader device, our developed system is portable, accurate, and convenient. Using an image processing technique, this application can replace a cumbersome reader device and effectively measure the presence of the analyte in a sample liquid in a quantitative way. Compared to the conventional technologies, our proposed method maintains its estimation performance under diverse lighting conditions without requiring an external LFA housing device. The main contributions of this paper are summarized as follows:

- A convenient and portable alternative to read LFA strips using solely a smartphone. The conventional techniques require external devices. Our proposed method uses a novel smartphone application that instantly detects an analyte quantity in an LFA strip without any prior calibration or any requirement of an external device.
- Resilience at various light conditions in detecting the analyte amount in an LFA strip. The performance of existing techniques is affected by the environment such as lighting conditions. Our proposed algorithm can accurately estimate the quantity of the analyte in an LFA strip in diverse lighting conditions.

## 2. Materials

### 2.1. Input Data: LFA Pads

**Figure 1:**
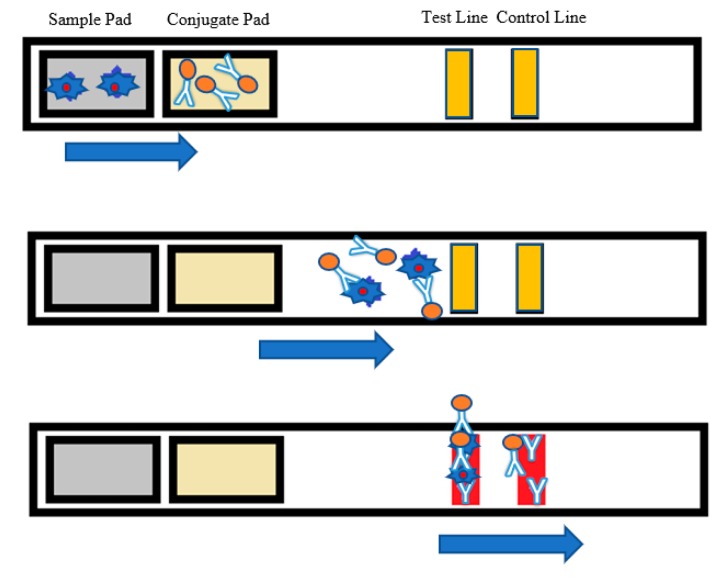
Lateral flow assay (LFA) strip architecture where the analyte is detected in the test line, and the red control line indicates that the test was performed.

An LFA strip contains four primary parts—sample pad, conjugate pad, membrane, and absorbent pad [31]. The sample pad is the region where the sample solution is distributed. The sample solution is designed to flow from the sample pad to the conjugate pad, which contains the conjugate labels (i.e., gold nanoparticles) and antibodies. Figure 1 shows the architecture and working principle of an LFA strip. The conjugate tags bind to the analyte molecules of the sample solution and flow along the strip. The membrane is made of nitrocellulose material. As the sample moves along, reagents situated on the test lines of the porous membrane capture the conjugated labels bound to the target analyte. The control line on the membrane consists of specific antibodies for the particular conjugate and captures the conjugate tags. These phenomena are observed as the red color in the test/control lines. The color intensity of the test line is expected to vary proportionally to the quantity of the analyte in the test sample. However, there is no color change in the test line if the target analyte is absent in the sample. A colored control line assures that the test has been performed well [32], as shown in Figure 1.

**Figure 2:**
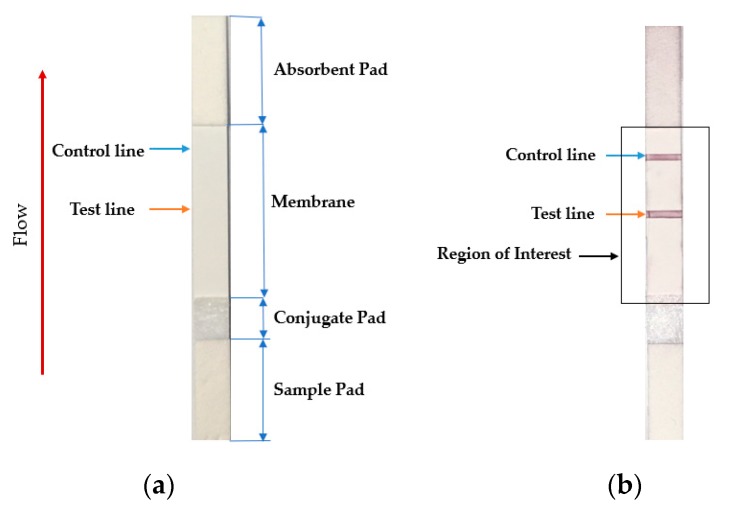
(**a**) Sample LFA strip and (**b**) region of interest of an LFA strip with an analyte. The intensity and density of red color in the test line region determine the amount of analyte in the sample.

The analyte quantity in the sample is expected to be proportional to the intensity of the red colored test line region. Hence, a weighted summation of red color in the test line region can be considered as the parameter for the detection of analyte quantity, where the weight is considered to be the color intensity of each pixel. Figure 2a shows a sample LFA test strip with its consisting parts, while Figure 2b shows the ROI (see rectangular box) and the test and the control lines (see orange and blue lines). 

### 2.2. Data Acquisition Using a Smartphone Camera

**Figure 3:**
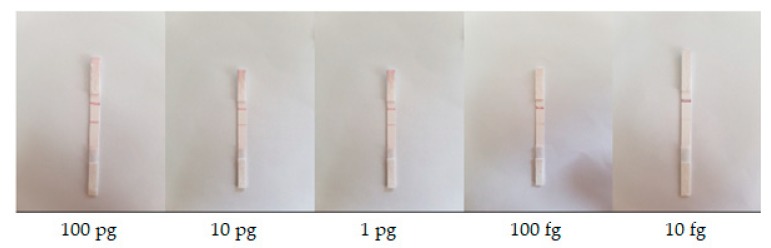
LFA strips for different quantities of analyte under laboratory ambient lighting condition.

LFA strips of known quantities of target analyte on sample pads were obtained from the Korea Research Institute of Bioscience & Biotechnology (KRIBB). The target analyte was albumin protein (albumin from human serum acquired from Sigma Aldrich, Saint Louis, MO, USA [33] with product number A9511), and the conjugate tags were gold nanoparticles with an albumin antibody. The solvent of pH 7.4 PBS (phosphate buffered saline, purchased from Gibco, Paisley, UK) was used to prepare 10 microliters (μL) of solution of albumin sample. Here, we considered the following five different concentration levels: 10 ng/mL, 1 ng/mL, 100 pg/mL, 10 pg/mL, and 1 pg/mL. Three sets of samples were available for the study, with each set containing five LFA strips having five different concentrations. As a result, the corresponding analyte quantities of five different concentration levels are 10 ng/mL × 10 μL = 100 pg, 1 ng/mL × 10 μL = 10 pg, 100 pg/mL × 10 μL = 1 pg, 10 pg/mL × 10 μL = 100 fg, and 1 pg/mL × 10 μL = 10 fg, respectively. An Institutional Review Board (IRB) assessment was not performed because the sample was purchased. Figure 3 shows sample images of LFA set #1. Each reading was taken five times to reduce error. Hence, a total of 15 LFA strips were used, and 75 readings were obtained in this study. Two sets, each consisting of five LFA strips of the aforementioned quantities (set #1 and #2), were used to train the machine learning model, and the other set was used for validating and testing the model of the proposed method. The data acquisition procedure was performed with an Android smartphone Samsung Galaxy S7 Edge, Samsung Electronics, Seoul, South Korea [34] with Android version 8.0.0. We used the rear camera of the smartphone, which had a maximum resolution of 12 megapixels (4032 × 3024 pixels). Autofocus function was enabled, and the flashlight was turned off during the data acquisition procedure. Images of the LFA strips were captured under different ambient lighting conditions in the laboratory. The room had a floor area of 41.475 m^2^ and was illuminated with a 36-Watt ceiling light and a 27-Watt table lamp, respectively. The method was evaluated based on acquired data using MATLAB 2018b, The MathWorks, Inc., Natick, Massachusetts, USA [35]. 

## 3. Methods

### 3.1. Feature Extraction 

We considered the number of red pixels accumulated on the test line as a feature. We assumed that the cumulative sum of red pixels’ intensities in the test line varied proportionally with analyte quantity under a specific lighting environment. To extract this feature, we performed the following: (1) selecting the test line region and the control line region, (2) segmenting image via optimal thresholding using Otsu’s method [36], and (3) calculating weighted sum of red pixels’ intensities, which are explained in Section 3.1.1, Section 3.1.2, and Section 3.1.3, respectively. During all these steps, image preprocessing and machine learning techniques were applied as described in these sections.

#### 3.1.1. Test and Control Line Region Selection

**Figure 4:**
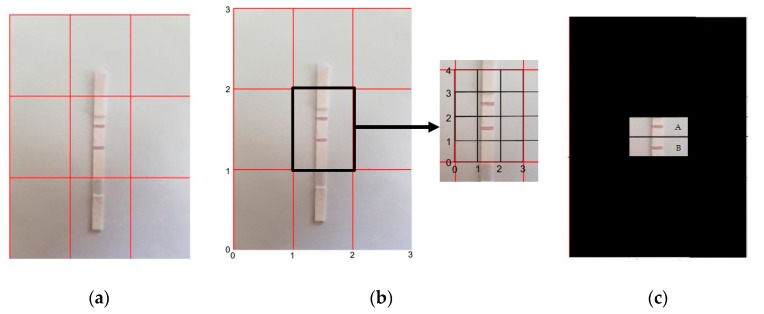
(**a**) A 3 × 3 grid of the smartphone camera view with proper positioning of LFA, (**b**) dimension extraction of regions from the center box ((1, 1), (2, 1), (1, 2), (2, 2)), with the test line and the control line position indexes ((0, 1), (3, 1), (0, 2), (3, 2)) and ((0, 2), (3, 2), (0, 3), (3, 3)), respectively, and (**c**) the control line and the test line regions (marked as A and B, respectively).

To extract the test and control line regions, the acquired image is cropped with fixed dimensions and a fixed aspect ratio [37]. This technology has been readily available in current generation cellular devices, and the procedure has been used in printing, extracting thumbnails, and framing digital pictures. Accurate placement of the LFA strip under the camera is required for exact approximation of analyte quantity. Figure 4a shows an example of an accurate placement of an LFA under a smartphone camera, where the ROI sits exactly in the center box of the 3 × 3 grid of the camera view. Then, the center grid region’s dimensions are readily extracted from the acquired LFA image. Figure 4b shows the indexes of the center box’s boundary points: ((1, 1), (2, 1), (1, 2), (2, 2)). The developed application automatically crops and extracts the test and control line regions from the center grid point locations of the image. The regions are obtained from inner grid indexes for the test line ((0, 1), (3, 1), (0, 2), (3, 2)) and the control line ((0, 2), (3, 2), (0, 3), (3, 3)). As shown in Figure 4c, the control line and the test line are identified as region A and region B, respectively. These regions are then considered for further calculation.

#### 3.1.2. Image Segmentation via Optimal Thresholding Using Otsu’s Method

**Figure 5:**
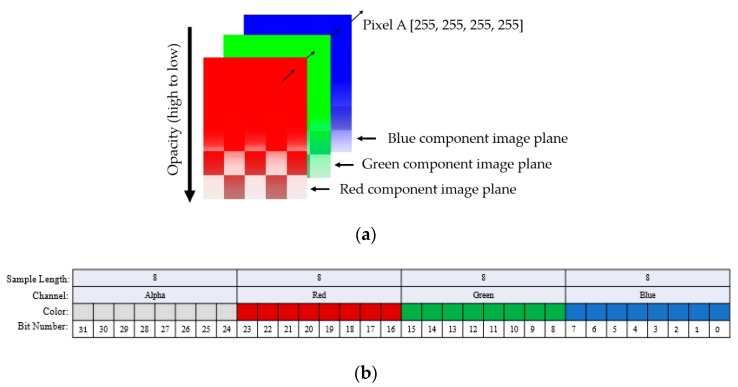
Representation of an image obtained using a Samsung Galaxy S7 smartphone. (**a**) Alpha-Red-Green-Blue (ARGB) representation of an image. An ARGB image consists of four planes, which are the alpha, red, green, and blue channels. Each channel’s intensity value can vary from 0 to 255. A pixel in the original image shows the combination of the intensity values of the color channels, and (**b**) the 32-bit representation of a pixel of the image (**a**). In an ARGB image, each pixel corresponds to a specific 32-bit binary value, of which the lowest eight bits represent blue, the next 8 bits represent green, the next eight bits for red, and the highest eight bits represent the alpha channel intensity values. The pixel’s color is the combination of these color intensities.

As mentioned in Section 2, we captured the LFA strip images using a Samsung Galaxy S7 smartphone camera with the resolution of 12 megapixels (4032 × 3024 pixels). The Samsung Galaxy S7 camera generated LFA images in Alpha-Red-Green-Blue (ARGB) 32-bit color space consisting of alpha (A), red (R), green (G), and blue (B) channels. Here, the A, R, G, and B channels represent opacity and red, green, and blue intensities, respectively. Figure 5a,b show the ARGB representation of an image and the 32-bit representation of a pixel in the image, respectively. Each pixel can have 2^32^, or 4,294,967,296, different representations since a pixel is represented by 32 bits. 

**Figure 6:**
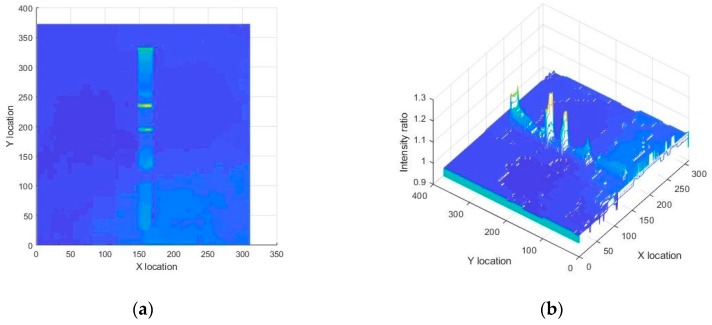
Ratio of red channel to green channel of a sample LFA strip is visualized by (**a**) a 2D surface, and (**b**) a 3D surface. It is clear that the test and control lines had the maximum value that can be used to differentiate them from other regions.

Denoting the red color intensity by $I_{R}\left(x,\;y\right)$ and the green color intensity by $I_{G}\left(x,\;y\right)$, we observed that the ratio of red to green channel intensity $\left(I_{R}\left(x,\;y\right)/\;I_{G}\left(x,\;y\right)\right)$ was noticeably different near the test/control line regions compared to other regions as shown in Figure 6. Specifically, $I_{R}\left(x,\;y\right)\;/\;I_{G}\left(x,\;y\right)$ values for pixels of an LFA strip image were nearly unity except for the test and control line regions.

Otsu’s multilevel thresholding method [36,38] has been included in this paper, which thresholds an image automatically by minimizing variance of intra-class intensity level. This method was applied to the extracted control line to obtain an optimal threshold value $\left(TH_{\mathrm{mask}}\right)$ for masking. This masking is used to only consider the ROI for calculation. This threshold value was applied to the whole image for creating the mask, which consists of points whose red to green channel intensity ratio values are higher than $TH_{\mathrm{mask}}$. Denoted by $I_{\mathrm{mask}}\left(x,\;y\right),$ the mask intensity value at the pixel location (x, y), $I_{\mathrm{mask}}\left(x,\;y\right)$ was calculated by the following equation:

(1) $$I_{\mathrm{mask}}\left(x,\;y\right)\;=\{\begin{array}{ll} 1, & \mathrm{if}\;\;\frac{I_{R}\left(x,\;y\right)}{I_{G}\left(x,\;y\right)}\;>TH_{\mathrm{mask}},\\ 0, & \mathrm{otherwise}. \end{array}$$

**Figure 7:**
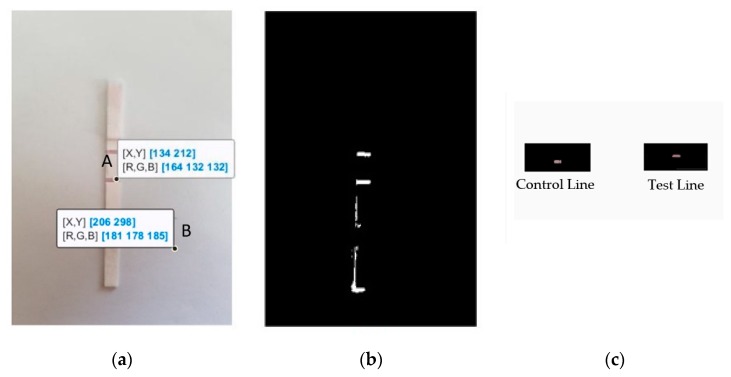
(**a**) Visualization of two pixels in a sample LFA strip image with red, green, and blue channel intensity values. Pixel A is in the test line region and pixel B is in the non-ROI region, (**b**) the calculated mask where the red to green intensity ratio was greater than the threshold *TH*_mask_, and (**c**) the extracted control line and test line regions are shown.

Figure 7a has been obtained from MATLAB to visualize the color difference between the ROI and the non-ROI region. In the test and the control line regions of the image, the red intensity values (in the range near 160) are larger than the green intensity values (in the range near 130), while the other regions had comparable red and green intensity values. Evidently, the intensity ratio of red to green color channels showed a higher difference in the test and control line regions, i.e., a higher $I_{R}\left(x,\;y\right)/I_{G}(x,\;y$) value. Using the optimal threshold (*TH*_mask_), the mask image was obtained, which is shown in Figure 7b. Figure 7c shows the cropped final image of the selected control line and test line regions.

#### 3.1.3. Calculation of the Weighted Sum of Red Pixels’ Intensities

**Figure 8:**
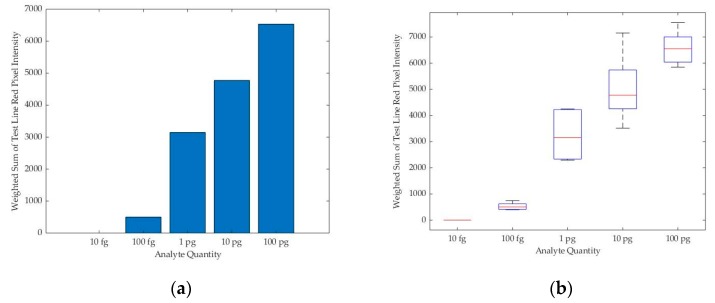
(**a**) Bar chart for the median of the readings for the weighted sum of the test line red pixels’ intensity values against the quantity of analyte present in the sample in a specific lighting condition; (**b**) the boxplot for multiple readings for each analyte quantity.

We observed that the sum of color intensities of the red pixels on the test line region tends to increase as the analyte quantity increases under a specific lighting environment, as shown in Figure 8. From this observation, we assumed that the counted red pixels’ intensity values on the test line from the captured smartphone camera image can estimate the analyte quantity from the LFA strip. 

Equation (2) shows the method for calculating the weighted summation of red pixels’ intensity values in a region (*S*_region_).

(2) $$S_{\mathrm{region}}=\sum I_{R}\left(x,y\right).$$

This equation is applied throughout the extracted control and test line. By calculating the weighted summation of the red pixels’ intensity values of the control line and test line regions *S*_control_ and *S*_test_ were obtained from the masked image.

### 3.2. Multiclass Classification Using MachineLearning Techniques

#### 3.2.1. Input Parameter for Classification (Test to Control Line Signal Intensity (T/C) Ratio)

**Figure 9:**
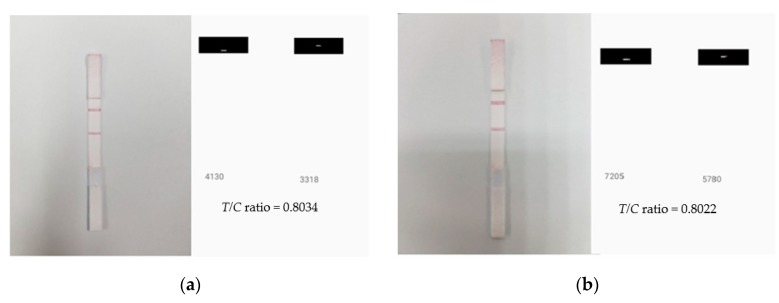
LFA strip and calculated *T/C* ratio for two different lighting conditions: (**a**) 36-Watt LED ceiling lamp and (**b**) 27-Watt LED table lamp illuminated environments, showing a similar *T/C* ratio.

The proposed method utilized a regression analysis [39,40] to approximate the analyte quantity and predict the value using machine learning techniques. As an input parameter for regression analysis, we considered the ratio of the test to control line signal intensity (*T/C* ratio), since the *T/C* ratio increase proportionally with analyte quantity despite of variation in illumination. The *T/C* ratio is expressed in Equation (3),

(3) $$TC\mathrm{ratio}\;=\;\frac{S_{\mathrm{test}}}{S_{\mathrm{control}}},$$

where *S*_test_ and *S*_control_ are the weighted sums of red pixels’ intensity values of the test and control lines, respectively. In this paper, we observed that the *T/C* ratio remained stable in different luminous environments for the same analyte quantity. Figure 9 shows two readings of the same LFA strip under two different ambient light environments. To simulate these two different lighting environments, a 36-Watt ceiling light and a 27-Watt table lamp light was used in a laboratory of 41.475 m^2^ floor area (see Section 2 for setup). The *T/C* ratios obtained had 0.15 percentage difference, which validates its ability as a classifier parameter since it is resilience at different light conditions.

The *T/C* ratio from different readings of available LFA sets (mentioned in Section 2): set #1, set #2, and set #3 are shown in Table 1, Table 2 and Table 3.

#### 3.2.2. Regression and Classification

**Figure 10:**
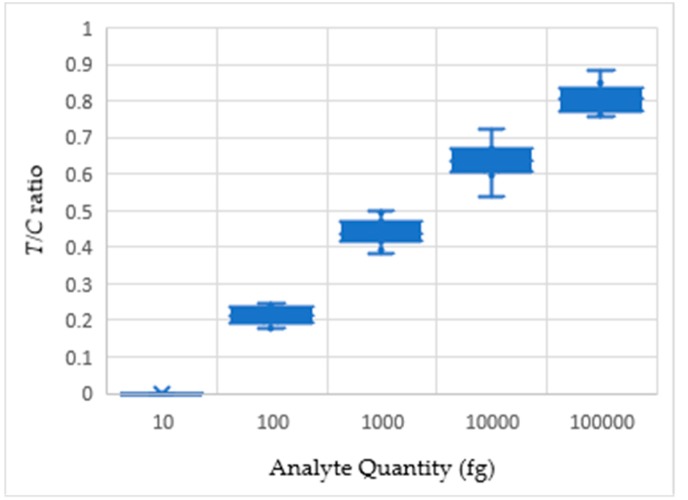
Analyte quantity against *T/C* ratio plot for three different LFA sets.

Visual detection methods, which has been used to detect analyte quantity, are qualitative and erroneous due to absence of quantification parameters. From the conjugation of albumin with gold nanoparticle tags, a comparative valuation of quantifiable color density is possible. Figure 10 shows the box whisker plot of the acquired *T/C* ratios for different readings.

A regression analysis was performed based on the feature parameter (*T/C* ratio) from Table 1, Table 2 and Table 3. The approximations obtained from the regression analysis were utilized for classification into the categories mentioned in Section 2.2 using machine learning technique. A linear support vector machine (SVM) classifier [41] was adopted for the classification of analyte quantity categories. The input parameter for the SVM is the approximated quantities obtained from the regression analysis. After training the linear SVM classifier using two training LFA sets with known analyte quantities, the trained classifier estimates the analyte quantity of a test LFA set.

### 3.3. Developed Smartphone Application

**Figure 11:**
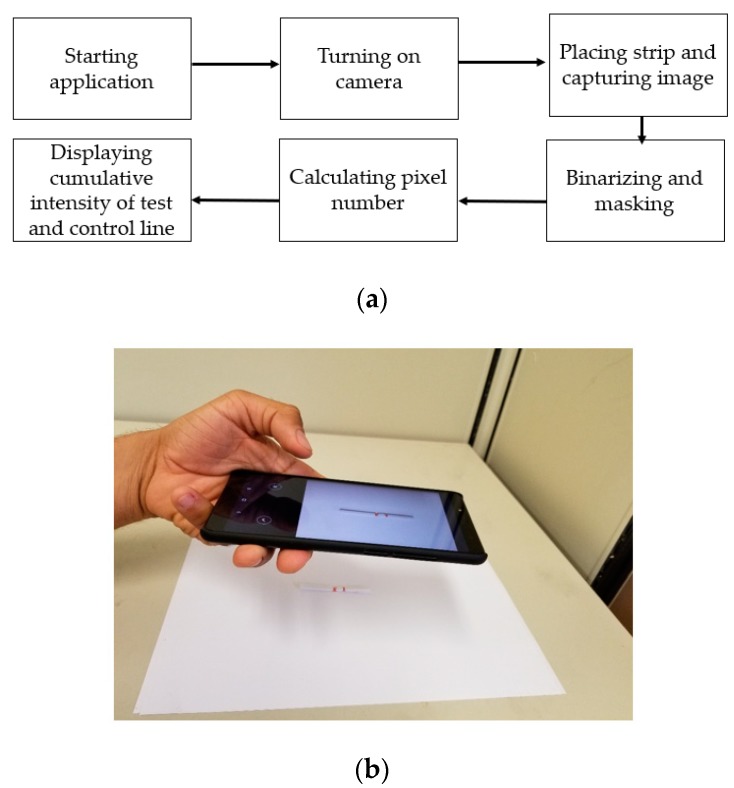
(**a**) The flowchart describing the subsequent operations of our developed application; (**b**) the data acquisition procedure with accurate placement using our developed application.

A smartphone application was developed on the Android platform following the proposed method described in Section 3.1 and Section 3.2. Figure 11a shows the flowchart of our developed application’s operation, and Figure 11b shows an example of data acquisition using our developed application. 

**Figure 12:**
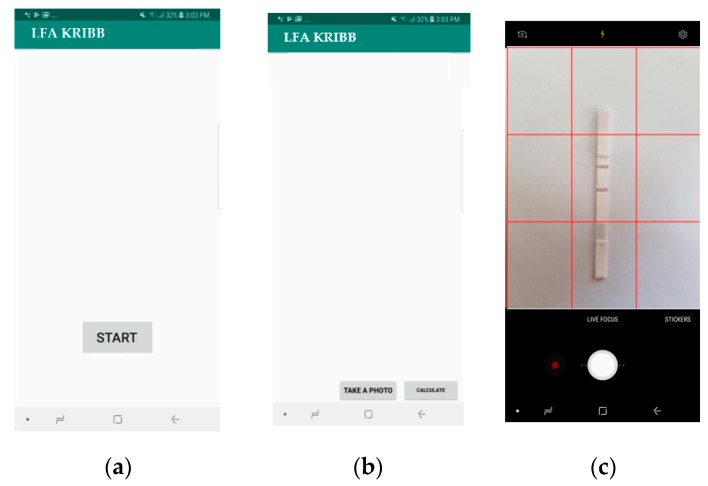

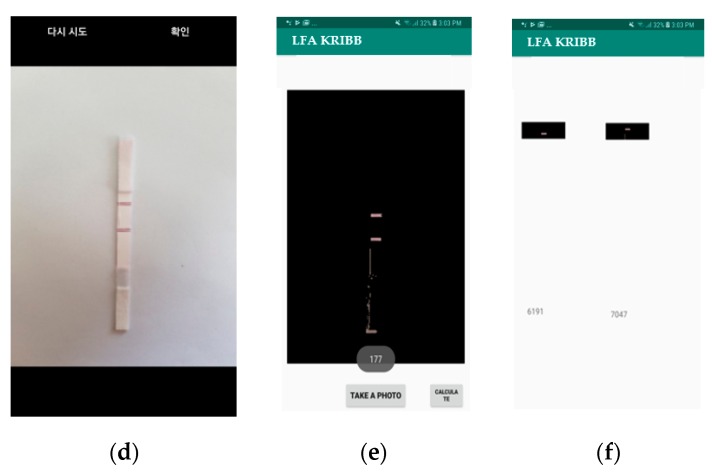
Proposed application for the detection of an analyte in an LFA strip: (**a**) home screen, (**b**) second screen with an image capture option, (**c**) camera view with a 3 × 3 grid spacing, (**d**) captured raw image, (**e**) processed image using the created mask (total pixel number shown at the bottom of the image), and (**f**) calculated control (left) and test (right) line weighted sum of pixels’ intensity values.

The smartphone application was tested on a sample LFA to evaluate the performance in a real-life scenario. The testing procedure was as follows: a test strip was placed under the smartphone on a white background. Using the grid view of the camera, the ROI was positioned inside the center box. The image was captured, and the ROI was obtained. The application then created a mask using the preprocessing technique mentioned in the proposed algorithm. In the masked region, the weighted sums of red pixels’ intensities of the test and the control line regions were calculated. Then, *T/C* ratio values that corresponded to the quantity of the analyte were calculated. Figure 12 shows the application screens and steps of operation of an LFA strip with a 100 pg analyte quantity. Figure 12c shows the positioning of the LFA strip in the center box of the grid view. Figure 12e shows the test and control line with the number of pixels in the mask, and Figure 12f shows the weighted sums of red pixels’ intensities of the control line (6191) and test line (7047). 

**Figure 13:**
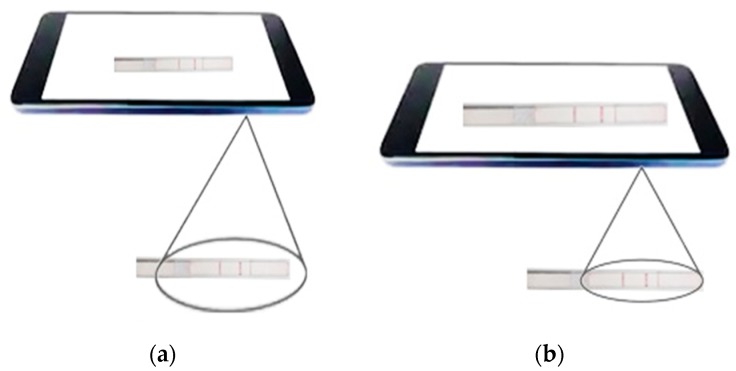
(**a**) Exact placement of the LFA strip under a smartphone camera and (**b**) an improper placement of the LFA strip—a larger field of view due to the proximity of the camera to the LFA strip.

For accurate estimation of the quantity of the analyte, it is essential to detect the ROI precisely. Since mobile devices do not have a fixed position with respect to the test strip, smartphone-based measurement is usually weak and prone to motion blur artifacts [12]. Moreover, due to the inherent unstable nature of the human hand (i.e., movement and shaking) and lack of a stable supporting device (i.e., tripod), the placement of the camera on the exact ROI is difficult, causing the measurement to be erroneous [42]. Hence, for an accurate comparison analysis, the camera must be placed at a certain distance from the test strip covering the ROI for each of the test cases. Figure 13 compares correct (see Figure 13a) and incorrect placements (see Figure 13b) of an LFA strip under a smartphone camera. As shown in Figure 13b, the larger field of view can lead to errors in calculating the number of pixels in the ROI.

**Figure 14:**
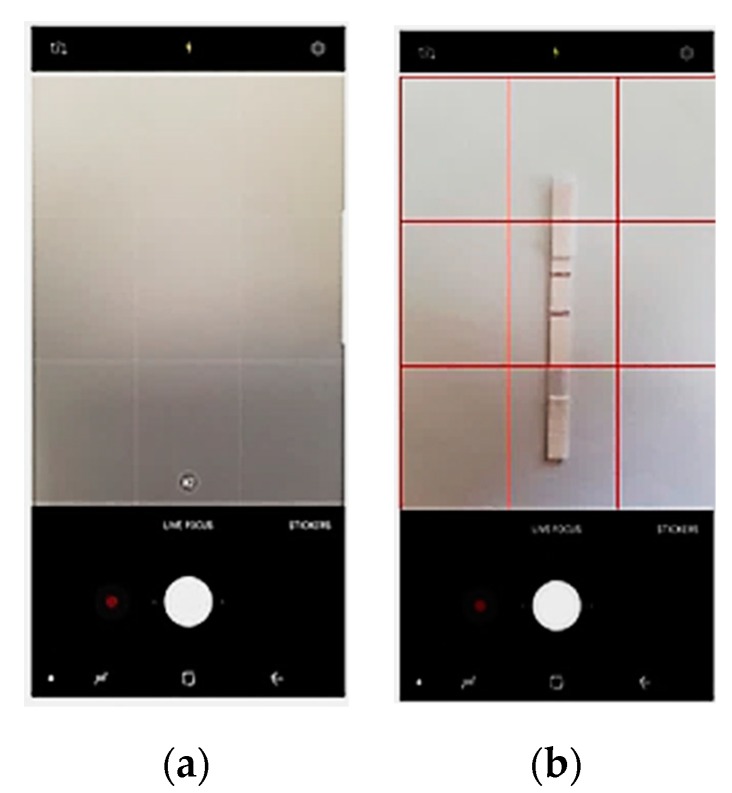
(**a**) Android smartphone (Samsung Galaxy) camera view with a 3 × 3 grid positioning and (**b**) LFA strip ROI positioned in the center box.

In order to counter the challenge of accurate placement, we implemented an additional function in our developed application which helps users to place the strip in a correct position using grids, as shown in Figure 14. Specifically, the application shows a 3 × 3 grid view on the screen and prompts the user to set the control line and test line in a certain position inside the center grid box, as shown in Figure 14b. Thus, the reference distance is fixed for all experiments. To reduce the error due to motion blur artifacts, five readings were taken for each case.

## 4. Results

The proposed method first approximates the analyte quantities using the regression analysis, and then predicts the quantities using the machine learning classifier, as described in Section 3. Section 4.1 explains the performance metrics to evaluate the regression and classification performance of the proposed method. Then, the regression and classification performance are evaluated in Section 4.2. Section 4.3 shows the smartphone application capability in terms of functionality and efficiency. 

### 4.1. Performance Metrics

The regression analysis of the proposed method shows the relationship between the analyte quantity and the *T/C* ratio for the acquired readings (mentioned in Section 3.2). The calibration curve, which can be used to approximate the unknown quantity of analyte in the sample, was obtained from the regression analysis. From the curve, the *R*^2^ value and the standard error of detection (σ) were calculated according to Equations (4) and (5), respectively.

(4) $$R^{2}=\frac{SS_{\mathrm{residual}}}{SS_{\mathrm{total}}},$$

where *SS*_residual_ is the measure of the discrepancy between the data and the estimation model, and *SS*_total_ is the sum of the squares of the difference of the obtained data and its mean, and

(5) $$\sigma =\frac{\sqrt{\sum {\left(Y-Y^{\prime }\right)}^{2}}}{N},$$

where *Y* is an actual data, *Y’* is a predicted data, and *N* is the number of acquired data points. The *R*^2^ value shows the statistical measure of proportion of variance, whereas σ measures the precision of the sample mean of detection. Moreover, the performance of the regression is evaluated in terms of the following metrics: limit of detection (*LOD*), limit of quantification (*LOQ*), and coefficient of variation (*CV*) [43,44]. *LOD* is the lowest possible concentration of analyte which can be detected with reliability and separated from the limit of blank (*LOB*). *LOB* is represented as the maximum quantity of analyte measured when no analyte is present in the sample. *LOQ* is the lowest quantity of analyte detected with a high confidence level. *LOQ* is the performance metric for the method or measurement system. *CV* is the relative variability calculated from the ratio of the standard deviation to the mean. *LOD*, *LOQ*, and *CV* are calculated according to Equations (6)–(8), respectively.

(6) $$LOD=LOB+1.645\times \frac{\sigma }{m},$$

(7) $$LOQ=10\times \frac{\sigma }{m},$$

where *σ* is the standard error of detection, and *m* is the slope of the calibration curve;

(8) $$CV=\frac{s}{\mu },$$

where *s* is the standard deviation of the readings, and *μ* is the mean of the readings for each analyte quantity.

On the other hand, the performance of the classifier is evaluated in terms of accuracy, which is defined as follows:

(9) $$Accuracy=\frac{TP+TN}{TP+TN+FP+FN}\times 100\%,$$

where *TP*, *FP*, *TN*, and *FN* are the numbers of true positives, false positives, true negatives, and false negatives, respectively.

### 4.2. Regression and Classification Results

**Figure 15:**
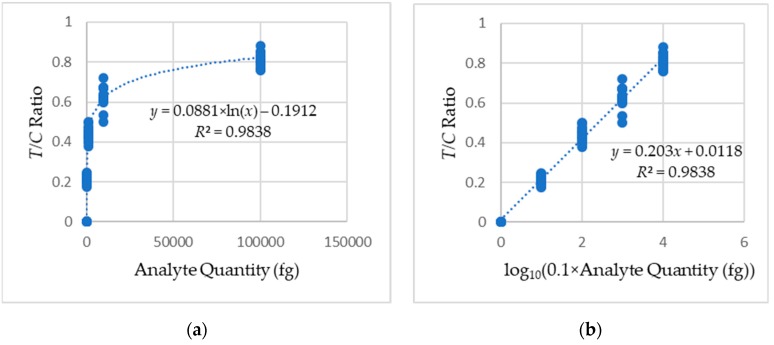
Calibration curves obtained from the regression analysis of the LFA sets: (**a**) logarithmic and (**b**) linear.

Figure 15 shows the regression analysis of the analyte quantity against *T/C* ratio. The calibration curve has an equation in the form: *y* = 0.203*x* + 0.0118, where *x* is the analyte quantity, and *y* is the *T/C* ratio. The obtained *R*^2^ value of 0.9838 from Equation (4) shows a strong correlation between the readings and the fitted calibration curve. The standard error of detection (σ) value of 0.007 was calculated from Equation (5), and the slope of the calibration curve (*m*) of 0.203 was obtained from the calibration curve. The limit of detection (*LOD*) of 0.000026 nM (1.71 pg/mL) and the limit of quantification (*LOQ*) of 0.00039 nM (26.48 pg/mL) were calculated using the values of σ and *m* according to Equations (6) and (7), respectively. The coefficients of variation (*CV*) obtained from calculations using Equation (8) are shown in Table 4. 

A linear SVM classifier with a five-fold cross validation was trained using the approximated quantities obtained from the two training LFA sets. Each set contained the following analyte quantities: 10 fg, 100 fg, 1 pg, 10 pg, and 100 pg. The confusion matrix for the training sets is shown in Table 5. The SVM decision boundary obtained from the training sets was applied to the test LFA set. Each test strip result was evaluated 10 times. The confusion matrix for the testing set is shown in Table 6. 

The analyte quantity detection accuracy of the proposed method was calculated to be 98%, according to Equation (9). The performance metrics for the classifier show that our smartphone application could detect four classes out of five with 100% accuracy. 

### 4.3. Smartphone Application Capability 

**Figure 16:**
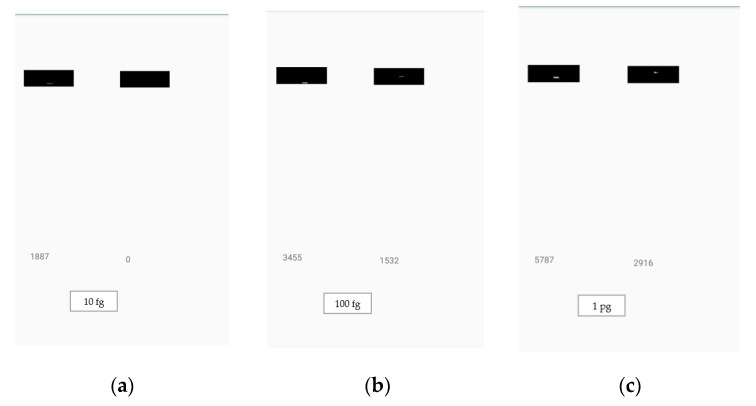

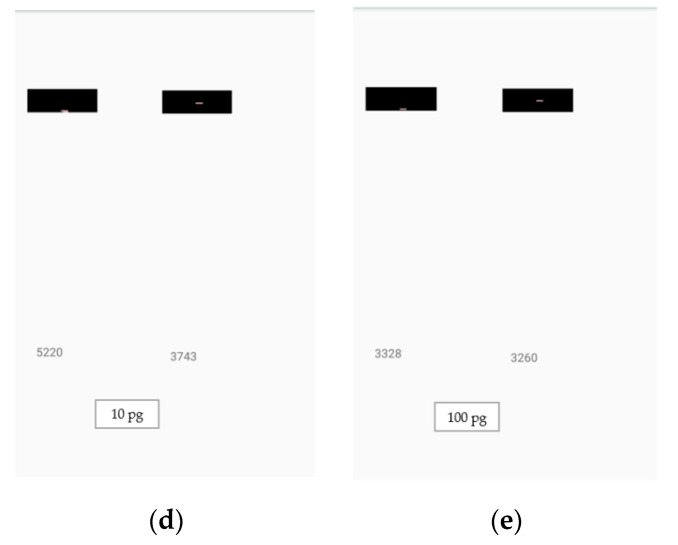
Estimation of analyte quantity: (**a**) 10 fg, (**b**) 100 fg, (**c**) 1 pg, (**d**) 10 pg, and (**e**) 100 pg, using the developed application. The smartphone application was used for the detection of analyte quantities under various lighting conditions. The number on the left is the control line weighted sum of red pixels’ intensities, the number on the right is the test line weighted sum of red pixels’ intensities, and the analyte quantity is displayed at the bottom of the screen.

Figure 16 shows the screen captures of five readings of a sample set of LFA strips from our application. The proposed quantitative method was able to detect the analyte quantity instantly after it was trained using the SVM. Training of the proposed method required 0.817 s using a cloud computer equipped with Windows 64-bit, Intel Core i7-6700 CPU @ 3.40 GHz (eight CPUs), 16,384 MB Ram, and MATLAB (2018b) programming.

## 5. Discussion

Ruppert et al. [45] compared their method to the method used by the *ImageJ* software [46,47], which is a well-known open platform tool for scientific image analysis. Similarly, we compared our proposed method to the *ImageJ* software’s method [46,47]. For a fair comparison, Otsu’s thresholding, which is included in our method, was also included in the *ImageJ* software. 

Table 7 compares the performance of the proposed method and the method used by *ImageJ* software under ambient lighting condition (mentioned in Section 2). The proposed method shows 2.96 times lower LOD and 798.92 times lower LOQ than the *ImageJ* method, which indicate that the performance of the proposed method was better at quantifying the analyte quantity. 

Table 8 compares our proposed method to the conventional methods in terms of functionality. Our method provides a more convenient, portable, robust, and inexpensive solution compared to the existing methods. Moreover, this method has a strong advantage in that only a smartphone is needed to accurately detect the amount of analyte without any additional equipment. 

## 6. Conclusions

In this paper, we have explored the possibility of using a smartphone application to detect the analyte quantity in a sample from an LFA. Using a novel image processing technique and SVM algorithm, we have proposed an automated smartphone application–based method for the detection of analyte quantity in LFA with acceptable accuracy. Our proposed smartphone application can successfully measure the analyte quantity in a sample from a set of LFA strips of analyte quantity varying from 10 fg to 100 pg (1 pg/mL to 10 ng/mL of concentration for 10 uL solution). An overall accuracy of 98.00% in the detection of unknown analyte quantities validated the promising performance of our application, which provides a convenient and rapid LFA reading. Our proposed method also supports the ubiquity (i.e., independent operability under a range of external lighting conditions), portability (i.e., no external device is required), efficiency (i.e., the method can determine the amount of analyte readily), and reliability (i.e., the proposed method achieved a reasonable accuracy in the test case) of our application. It can replace existing methods for the low-cost detection of LFA analytes in different application fields.

## Figures and Tables

**Table 1:** Calculated *T/C* ratio for LFA set #1.

Test Class	Reading 1	Reading 2	Reading 3	Reading 4	Reading 5
100 pg	0.764	0.782	0.772	0.758	0.762
10 pg	0.672	0.618	0.502	0.536	0.596
1 pg	0.434	0.404	0.394	0.452	0.380
100 fg	0.21	0.234	0.20	0.18	0.194
10 fg	0	0	0	0	0

**Table 2:** Calculated *T/C* ratio for LFA set #2.

Test Class	Reading 1	Reading 2	Reading 3	Reading 4	Reading 5
100 pg	0.806	0.807	0.82	0.85	0.836
10 pg	0.634	0.622	0.674	0.67	0.668
1 pg	0.456	0.456	0.438	0.472	0.426
100 fg	0.246	0.242	0.212	0.226	0.176
10 fg	0	0	0	0	0

**Table 3:** Calculated *T/C* ratio for LFA set #3.

Test Class	Reading 1	Reading 2	Reading 3	Reading 4	Reading 5
100 pg	0.796	0.828	0.882	0.64	0.798
10 pg	0.634	0.61	0.608	0.722	0.64
1 pg	0.426	0.5	0.496	0.416	0.476
100 fg	0.238	0.204	0.208	0.194	0.22
10 fg	0	0	0	0	0

**Table 4:** Mean, std. deviation and coefficient of variation of test to control line signal intensity (*T/C*) ratios for each of the analyte quantities of the LFA sets.

Analyte Quantity	Mean *T/C* Ratio	Std. Deviation of *T/C* Ratio	*CV* (%)
100 pg	0.791502	0.055011	6.950253
10 pg	0.624684	0.055213	8.838581
1 pg	0.440393	0.035544	8.070897
100 fg	0.211211	0.021829	10.33506
10 fg	0	0	0

**Table 5:** Confusion matrix for the training sets obtained by the linear support vector machine (SVM) model. The asterisk sign (*) denotes the misclassified analyte quantity for the training LFA sets.

	Predicted Class				
Actual Class	100 pg	10 pg	1 pg	100 fg	10 fg
100 pg	10	0	0	0	0
10 pg	1 *	9	0	0	0
1 pg	0	0	10	0	0
100 fg	0	0	0	10	0
10 fg	0	0	0	0	10

**Table 6:** Confusion matrix for testing the method with the SVM. The asterisk sign (*) denotes the misclassified analyte quantity for the test LFA set.

	Predicted Class				
Actual Class	100 pg	10 pg	1 pg	100 fg	10 fg
100 pg	10	0	0	0	0
10 pg	1 *	9	0	0	0
1 pg	0	0	10	0	0
100 fg	0	0	0	10	0
10 fg	0	0	0	0	10

**Table 7:** Performance comparison between *ImageJ* software and our proposed method.

	Proposed Method	*ImageJ* Method
LOD	0.000026 nM (1.71 pg/mL)	0.000077 nM (5.12 pg/mL)
LOQ	0.00039 nM (26.48 pg/mL)	0.31158 nM (20,720.4 pg/mL)

**Table 8:** Method comparison between existing methods and the proposed method.

	Ruppert et al. [45], Roda et al. [48], Balaji et al. [23]	Mokkapati et al. [49], RDS 2500 LFA Reader, Detekt Biomedical LLC. [25]	Preechaburana et al. [50]	Proposed Method
Uses smartphone	Yes	No	Yes	Yes
Requires external device	Yes	N/A	No	No
Works in varying lighting conditions	N/A	N/A	No	Yes
Approximates analyte quantity	Yes	Yes	Yes	Yes
Predicts based on machine learning	No	No	No	Yes
